# 120 Impact of Undergraduate Clinical Research Experience: Highlighting the UCLA Clinical and Translational Science Institute Research Associates Program (CTSI-RAP) – CORRIGENDUM

**DOI:** 10.1017/cts.2024.523

**Published:** 2024-04-23

**Authors:** Amanda Piring, Jim Morrison, Noah Federman, Laurie Shaker-Irwin, Sam Duong-Brett

The above abstract published with a duplication of author names. The correct author list is shown below.

Amanda Piring, Jim Morrison, Noah Federman, Laurie Shaker-Irwin and Sam Duong-Brett

University of California, Los Angeles

The original abstract has been corrected online to rectify these errors.
